# Clinical outcomes and cost-utility analysis of comprehensive geriatric assessment models in hospitalized frail older patients in Thailand

**DOI:** 10.1186/s12877-026-07718-x

**Published:** 2026-06-03

**Authors:** Patumporn Suraarunsumrit, Varalak Srinonprasert, Montarat Thavorncharoensap, Usa Chaikledkaew, Tanawan Kongmalai, Sutisa Pitiyarn, Ponnapa Petchthai, Kannika Tankayura, Chaninthorn Kliangda, Gareth McKay, Ammarin Thakkinstian

**Affiliations:** 1 Graduate Program, Mahidol University Health Technology Assessment (MUHTA), Mahidol University, Bangkok, Thailand; 2 Division of Geriatric Medicine, Department of Medicine, Faculty of Medicine, Siriraj Hospital, Mahidol University, Bangkok, Thailand; 3 Siriraj Health Policy Unit, Faculty of Medicine, Siriraj Hospital, Mahidol University, Bangkok, Thailand; 4 Social Administrative Pharmacy Division, Department of Pharmacy, Faculty of Pharmacy, Mahidol University, Bangkok, Thailand; 5 Division of Endocrinology and Metabolism, Department of Medicine, Faculty of Medicine, Siriraj Hospital, Mahidol University, Bangkok, Thailand; 6 Division of Geriatric Nursing, Department of Nursing, Faculty of Medicine, Siriraj Hospital, Mahidol University, Bangkok, Thailand; 7 Siriraj Academic Center of Geriatric Medicine, Faculty of Medicine, Siriraj Hospital, Transitional Co-Care Team, Mahidol University, Bangkok, Thailand; 8 Department of Nursing, Faculty of Medicine, Siriraj Hospital, Mahidol University, Bangkok, Thailand; 9 Centre for Public Health, School of Medicine, Dentistry, and Biomedical Sciences, Queen’s University, Belfast, Northern Ireland; 10 Department of Clinical Epidemiology and Biostatistics, Faculty of Medicine Ramathibodi Hospital, Mahidol University, Bangkok, Thailand

**Keywords:** Frailty, Comprehensive Geriatric Assessment, Transitional care, Quality of Life, Function, Readmission

## Abstract

**Background:**

Frailty increases vulnerability to adverse outcomes and healthcare utilization. Comprehensive geriatric assessment (CGA) improves these outcomes in high-income countries, but evidence in resource-constrained settings is limited. This study aimed to assess clinical outcomes and cost-effectiveness of two CGA models (CGA-ward; CGA-consult) compared to usual care in hospitalized frail older adults.

**Methods:**

Two data sources from Siriraj Hospital were utilized: A randomized controlled trial comparing CGA-consult with usual care in acute medical wards (September 2019 and March 2024), and a retrospective cohort of patients receiving CGA-ward care in an intermediate care ward (April 2023-June 2024). Outcomes included health utility measured by EQ-5D-5L, Barthel Index, and hospital readmissions over 6 months. Cost-utility analyses were conducted from a societal perspective. Multivariate mixed-effects regression with inverse probability of treatment weighting (IPTW) estimated incremental outcomes and costs for incremental cost-effectiveness ratios (ICERs).

**Results:**

Among 226 patients, 45 received CGA-ward, 89 received CGA-consult, and 92 received usual care. CGA-ward significantly improved utility scores (incremental mean: 0.259; 95% CI: 0.140, 0.378) and functional outcomes (mean difference: 37.94; 95% CI: 26.39, 49.48) compared to usual care. From a societal perspective, CGA-consult also showed a modest improvement in utility (incremental mean: 0.093; 95% CI: 0.007, 0.179). CGA-ward and CGA-consult provided US$937 and US$552 savings per patient, with QALY gains of 0.130 and 0.047, respectively. Estimated ICERs were -US$7,208 and -US$11,745 per QALY gained, representing cost-savings for both interventions compared to usual care.

**Conclusions:**

CGA-ward represents an intermediate care (IMC) model providing improved clinical and economic benefits, supporting its implementation in resource-limited settings. CGA-consult remains a viable alternative where ward-based care is not feasible, highlighting the value of integrating CGA into routine hospital care for frail older adults.

**Trial registration:**

The parent randomized controlled trial was registered with the Thai Clinical Trials Registry (TCTR20190910002) on 10 September 2019.

**Supplementary Information:**

The online version contains supplementary material available at 10.1186/s12877-026-07718-x.

## Introduction

 Frailty reflects a clinical manifestation of age-related decline across multiple systems that can lead to functional decline, disability [1–3], and reduced health-related quality of life (HRQoL) [4, 5]. The pooled prevalence of frailty among hospitalized older adults has been reported at approximately 41% (95%CI: 38.4, 44.4) [6] and is associated with adverse clinical outcomes, increased healthcare costs, and higher mortality [3, 7, 8]. Addressing and identifying effective interventions for frailty is crucial for supporting aging global populations.

Hospitalized frail older patients often require a comprehensive care approach in addition to acute medical treatments due to the complexity of their conditions. Comprehensive geriatric assessment (CGA) represents a systematic and holistic approach for older patients that assesses physical, functional, psychological, and social aspects that inform appropriate interventions to prevent unfavorable outcomes [9–11]. CGA programs are typically provided by a multidisciplinary team (MDT) and involve regular assessments pre- and post-interventions to optimize patient care [10, 12].

Two CGA models for the inpatient setting are widely implemented [13]. First, ***CGA-consult*** involves frail patients hospitalized in a non-geriatric, acute care ward following assessment by a MDT and recommendation of treatment plans according to CGA principles [14]. Second, ***CGA-ward*** involves frail patients hospitalized under the care of a physician-led team, commonly a geriatrician, supported by a MDT to provide comprehensive assessments and interventions for older patients in a dedicated ward [13].

However, most of the evidence supporting the benefits of CGA models comes from high-income countries [12, 14, 15], raising concerns about the applicability of their findings in resource-limited settings [16]. Moreover, some studies reported conflicting results regarding the cost-effectiveness of implementing CGA models [17, 18], highlighting the dependence of cost-effectiveness on healthcare settings, intervention designs, and economic perspectives.

In low- and middle-income countries (LMICs), healthcare systems often face constraints, including limited MDT capacity, insufficient geriatric training, and fragmented infrastructure. As a result, data from LMICs to assess these treatment options remains scarce [17, 18]. The only study from India showed the efficacy of transitional care post-discharge without analysis from an economic perspective [19]. To address this gap, our study compared the clinical and economic outcomes of three care models: CGA-consult, CGA-ward, and usual care for hospitalized frail older adults in a resource-constrained setting.

## Methods

### Study design

The study was a cost-utility analysis (CUA) conducted from a societal perspective to evaluate CGA interventions among hospitalized frail older patients at Siriraj Hospital, Thailand. Two sources of data were included. The first cohort used data from an interim analysis of a randomized controlled trial (RCT) comparing clinical outcomes between CGA-consult and usual care in acute medical wards (TCTR20190910002), with participant enrollment from September 2019 to March 2024. The second cohort consisted of a retrospective cohort of patients who received CGA-ward interventions in the intermediate care (IMC) ward between April 2023 and June 2024. Importantly, we used the clinical effectiveness of both CGA models derived directly from these cohorts within the same healthcare setting. This approach is appropriate to ensure that the CUA reflects real-world effectiveness and context-specific practice patterns. The study was approved by the Siriraj IRB under the following protocol numbers: Si 498/2019, Si 579/2023, and MU-MOU 669/2024.

### Inclusion and exclusion criteria

Inclusion criteria for both cohorts were hospitalized frail patients aged 60 years or older, who were admitted to the acute medical wards or dedicated IMC wards at Siriraj Hospital. Frailty was identified using the Thai version of the FRAIL Scale, which ranges from 0 to 5, with a score ≥ 3 defined as frail [20]. Frailty assessments were not performed in patients approaching the end-of-life, or required mechanical ventilation at admission, or resided in nursing homes. Frail older patients who were unable to complete follow-up assessments were excluded. To ensure independence between cohorts, research assistants verified patient identifiers and study records at the time of CGA-ward enrollment to confirm that no participants from the RCT (CGA-consult vs. usual care) were included in the CGA-ward cohort.

### CGA models versus usual care

#### CGA-consult

This cohort comprised frail older patients from the intervention arm of an RCT. They were assessed by a geriatrician-led MDT (nurse, pharmacist, dietician, physical therapist) who developed individualized plans targeting key geriatric syndromes and shared them with the primary team. The CGA in our study was delivered through a MDT approach, in which different domains were assessed by the responsible discipline at different time points on the same day and the information was shared via an electronic record used in the study. Specifically, in our model, cognition and social issues by nurses, medication review by pharmacists, nutritional status by dietitians, and functional status by physical therapists, while the geriatrician focused on medical and physical evaluation. Findings were subsequently integrated during multidisciplinary discussions or ward rounds to formulate individualized care plans. Post-discharge support for caregivers included 24-hour messaging, a 2-week follow-up call, and geriatrician-led teleconsultations at 1, 3, and 6 months (Appendix 1). We decided to take this approach to reflect on the possible model that should be implemented in our resource-limited settings.

#### CGA-ward

This cohort included frail older patients admitted to the IMC ward and managed by a geriatrician-led MDT (nurses, pharmacists, psychologists, Thai traditional medicine practitioners, social workers, dieticians, and therapists as needed). Interventions focused on family goal-setting and included problem prioritization, medication review, nutritional support, rehabilitation, and comprehensive discharge planning. Caregivers received hands-on training and preparation for home care. Outcomes were monitored through medical reviews and follow-up calls at 1, 3, and 6 months (Appendix 1).

#### Usual care

This cohort comprised the RCT control arm that received care based on acute medical needs. Geriatric and rehabilitation teams were consulted on request, and discharge planning was handled by the primary team without structured post-discharge care. Patients were followed by their primary physicians, with study outcomes assessed by the research team at 1, 3, and 6 months (Appendix 1).

### Data collection

Demographic data, comorbidities, and the Charlson Comorbidity Index (CCI) [21] were collected. Dementia status was defined using documented diagnosis and/or clinical evaluation by a geriatrician. Functional status was evaluated using the Barthel Index for activities of daily living (BADL) [22], where higher scores indicate better function. Nutritional status was assessed using the Mini Nutritional Assessment Short-Form (MNA-SF) [23].

HRQoL was measured using the EQ-5D-5 L instrument. Cost data were collected, including direct medical (DMCs) and direct non-medical costs (DNMCs).

### Clinical outcome assessments

The primary outcome was HRQoL assessed at 1, 3, and 6 months after enrollment by trained research assistants using the Thai version of the EQ-5D-5L questionnaire [24]. Responses were converted to a utility score using the Thai value set [25].

The number of participants contributing utility data varied across follow-up time points due to death, loss to follow-up, and a protocol modification during the study. Specifically, after the study had initiated, the follow-up period for participants in the RCT (CGA-consult versus usual care) was extended to 6 months. Consequently, some early-enrolled participants did not undergo prospective HRQoL assessments at later follow-up visits, particularly at 6 months, resulting in smaller sample sizes at this time point. In contrast, all participants in the CGA-ward group were enrolled after the protocol extension and completed the planned 6-month follow-up.

Secondary outcomes included the Barthel Index and readmissions at 1, 3, and 6 months. Readmission events were collected via patient or family telephone interviews after discharge, and by reviewing medical records.

### Cost measurements

Cost data were collected, including DMCs, DNMCs, and intervention-specific costs for comprehensive care provision. DMCs, including all hospitalization, readmissions, and outpatient or emergency visits, were estimated using a top-down approach based on routinely collected administrative data and converted using hospital-specific charge-to-cost ratios (2019–2023) from Siriraj Hospital. This approach was adopted to enable comprehensive cost capture in a real-world setting, where detailed patient-level resource utilization data were not consistently available. It also reflects actual expenditure patterns within the healthcare system.

DNMCs, including transportation, meals, accommodation, assistive devices, paid caregiving, and caregiver productivity loss, were collected through structured interviews at 1, 3, and 6 months post-discharge. Productivity loss was valued by the human capital approach using an hourly wage of THB 85.73, derived from Thailand’s 2023 Gross National Income per capita and exchange rate. Workforce costs were assessed by time-driven micro-costing of labor inputs from geriatricians and MDT members. No discounting was applied for the 6-month horizon. Costs were expressed in Thai Baht and converted to US dollars at the 2022 average rate (33.15 THB/US$).

### Statistical Methods

Sample size estimation was based on the population mean utility score of 0.58 (standard deviation (SD) 0.20) [26], with a minimally important difference (MID) of 0.09 [27]. Given the anticipated limited number of eligible participants receiving CGA-ward care, a 2:2:1 allocation ratio for usual care: CGA-consult: CGA-ward was applied. Assuming α = 0.05 and power = 0.80, the required total sample size was 205. Allowing for 10% loss to follow-up, the final target sample size was 225 participants (90, 90, and 45, respectively). Categorical variables were summarized as frequencies and compared using χ² tests, while continuous variables were expressed as mean ± SD or median (range) and compared with one-way ANOVA or Kruskal-Wallis tests, as appropriate.

To address baseline imbalances, inverse probability of treatment weighting (IPTW) was applied [28]. Propensity scores were derived from a multinomial logistic regression including relevant covariates (e.g., hypertension, diabetes, stroke, or transient ischemic attack (TIA) [11], chronic kidney disease (CKD) stage 3–5, dementia, baseline utility and function), with backward elimination retaining significant predictors. Balance was assessed using weighted standardized mean differences (MDs), with thresholds close to zero or ≤ 0.20 considered acceptable [29].

Outcomes were analyzed using IPTW. Continuous outcomes were assessed using mixed-effects linear regression, and dichotomous outcomes were assessed using mixed-effects logistic regression. Covariates significant at *p* < 0.1 were entered into multivariate models, with backward elimination retaining variables at *p* ≤ 0.05 plus clinically important factors (baseline utility, CKD stage 3–5, dementia). Potential outcome means and average treatment effects were estimated accordingly. Because the number of participants contributing outcome data varied across follow-up time points due to mortality and loss to follow-up, analyses were conducted using available-case data at each time point.

The incremental cost-effectiveness ratios (ICERs) were calculated using the standard formula: ICER = ΔAdjusted Cost ÷ ΔAdjusted QALYs. For the primary analysis, QALYs were derived by multiplying the adjusted utility scores by 0.5 to reflect the 6-month follow-up period, assuming a linear change in utility over time. Utilities were modeled using IPTW-adjusted mixed-effects regression, which provided stabilized estimates of incremental utility differences and, subsequently, incremental QALYs. Deaths during follow-up were explicitly accounted for in both utility and cost analyses. For QALY estimation, utility was assigned a value of zero at the follow-up time point at which death occurred. Cost data were also accumulated up to the time of death to ensure that mortality-related resource use was fully captured. Incremental costs were estimated using log-transformed ordinary least-squares regression, and adjusted costs were obtained by back-transforming the marginal means predicted from these models, enabling interpretation on the original cost scale [30]. All analyses were performed in STATA 17, with statistical significance defined at *p* < 0.05.

Uncertainty was assessed via probabilistic sensitivity analysis (PSA) with 5,000 Monte Carlo simulations in Excel. Parameter variability was incorporated by assigning probability distributions based on regression-adjusted incremental effects and corresponding standard errors. Gamma distributions were applied to adjusted incremental cost parameters to reflect skewness and non-negativity, while normal distributions were applied to incremental utility and QALY estimates based on regression-adjusted means and standard errors.

Simulations generated joint distributions of incremental costs and QALYs to characterize uncertainty around cost-effectiveness results. Incremental cost-effectiveness pairs were plotted on cost-effectiveness planes (CEPs), and cost-effectiveness acceptability curves (CEACs) were used to estimate probabilities across a range of willingness-to-pay (WTP) thresholds. A willingness-to-pay threshold of 160,000 Thai Baht (THB) per QALY (approximately US$ 4,826) was applied, consistent with thresholds recommended in the Thai health technology assessment guideline [31].

## Results

### Baseline characteristics

A total of 226 patients were included, comprising 92 patients in the usual care group, 89 in the CGA-consult group, and 45 in the CGA-ward group. At 6 months, mortality occurred in 10 patients (10.9%) in the usual care group, 13 patients (14.6%) in the CGA-consult group, and 1 patient (2.2%) in the CGA-ward group. Reasons for loss to follow-up are detailed in Appendix 2.

Baseline characteristics are shown in Table 1. Patients in the CGA-ward were older than those in the CGA-consult and usual care groups (82 vs. 77 vs. 76 years; *p* < 0.001) and had lower BMI (21.7 vs. 23.2 vs. 24.2 kg/m²; *p* = 0.030). Nutritional status tended to be poorer in the CGA-ward group, with lower mean MNA-SF scores (5.9 vs. 6.4 vs. 6.9), although this difference did not reach statistical significance (*p* = 0.056). The CGA-ward group had higher baseline dementia (55.6% vs. 15.7% vs. 8.7; *p* < 0.001) and stroke or TIA (57.8% vs. 25.8% vs. 25.0%; *p* < 0.001), but lower rates of diabetes (24.4% vs. 55.1% vs. 56.5%; *p* = 0.001), CKD stage 3–5 (22.2% vs. 62.9% vs. 65.2%; *p* < 0.001), and congestive heart failure (6.7% vs. 34.8% vs. 34.8%; *p* = 0.001). Baseline HRQoL (0.32 vs. 0.68 vs. 0.67; *p* < 0.001) and Barthel index (29.0 vs. 72.6 vs. 75.3; *p* < 0.001) were also significantly poorer in this group. Other baseline characteristics, including sex distribution, health benefit coverage, frailty score, CCI, coronary artery disease, and hypertension, were broadly comparable across groups.

**Table 1:** Baseline characteristics comparisons between groups of study participants

Characteristics	Usual care n=92	CGA-consult n=89	CGA-ward n=45	p-value
Age, year, mean (SD)	75.7 (7.8)	77.3 (8.6)	82.1 (8.7)	<0.001^a^
Female, n (%)	49 (53.3)	52 (58.4)	26 (57.8)	0.760
Health Benefit Coverage, n (%)				0.127
CSMBS	42 (45.7)	30 (33.7)	24 (53.3)	
UCS	24 (26.0)	30 (33.7)	14 (31.1)	
Other	26 (28.3)	29 (32.6)	7 (15.6)	
BMI, kg/m^2^, mean (SD)	24.2 (5.6)	23.2 (4.9)	21.7 (4.9)	0.030^a^
MNA-SF, mean (SD)	6.9 (2.5)	6.4 (2.4)	5.9 (2.6)	0.056
Nutritional status, n (%)				0.458
Normal	0 (0.0)	2 (2.2)	0 (0.0)	
Risk of malnutrition	34 (37.0)	32 (36.0)	14 (31.1)	
Malnutrition	58 (63.0)	55 (61.8)	31 (68.9)	
Frailty score, mean (SD)	3.4 (0.6)	3.5 (0.6)	3.4 (0.5)	0.948
Charlson comorbidity index, mean (SD)	6.6 (2.5)	6.5 (2.3)	6.7 (1.2)	0.902
Comorbidity, n (%)				
HT	83 (90.2)	76 (86.4)	38 (84.4)	0.573
Type2 DM	52 (56.5)	49 (55.1)	11 (24.4)	0.001^a^
CHF	32 (34.8)	31 (34.8)	3 (6.7)	0.001^a^
CAD	27 (29.4)	23 (25.8)	6 (13.3)	0.120
Stroke or TIA	23 (25.0)	23 (25.8)	26 (57.8)	<0.001^a^
CKD stage 3-5	60 (65.2)	56 (62.9)	10 (22.2)	<0.001^a^
Dementia	8 (8.7)	14 (15.7)	25 (55.6)	<0.001^a^
Barthel index score, mean (SD)	75.3 (24.6)	72.6 (26.4)	29 (22.7)	<0.001^a^
Utility score, mean (SD)	0.67 (0.28)	0.68 (0.25)	0.32 (0.26)	<0.001^a^
Principle diagnosis, n (%)				
Infection	36 (39.1)	33 (37.9)	7 (15.6)	0.014^a^
Non-infectious conditions	56 (60.9)	54 (62.1)	38 (84.4)	

*Abbreviations*: *BMI *Body mass index, *CGA *Comprehensive geriatric assessment, *CAD *Coronary artery disease, *CHF *Congestive heart failure, *CKD *Chronic kidney disease, *CSMBS *Civil Servant Medical Benefit Scheme, *HT *Hypertension, *MNA-SF *Mini Nutritional Assessment Short-Form, *TIA *Transient ischemic attack, *Type2 DM, *Type2 diabetes, *UCS *Universal Coverage Scheme, *SD *Standard deviation

^a^Indicates statistical significance (≤0.05)

Regarding principal diagnosis for admission, non-infectious conditions were more common among CGA-ward patients (84.4% vs. 62.1% vs. 60.9%; *p* = 0.014) (Table 1). In the usual care and CGA-consult groups, infections were the most frequent diagnoses (39.1% and 37.1%, respectively), particularly respiratory tract infections (3.3% and 12.4%) and urinary tract infections (12.0% and 6.7%). Among non-infectious conditions, cardiovascular conditions were predominant, including congestive heart failure (18.5% and 20.2%) and acute coronary syndrome (6.5% and 4.5%). In contrast, the CGA-ward group mainly comprised patients admitted for rehabilitation-related indications (84.4%), including post-orthopedic or surgical rehabilitation (44.4%), medical deconditioning (26.7%), and post-stroke rehabilitation (13.3%), reflecting the IMC setting (Appendix 3).

With respect to CGA with MDT, each group received a different proportion of interventions and a different approach (Appendix 4). Patients in the usual care group were not routinely managed under a structured CGA model. Only 3 of 92 patients (3.3%) received a formal geriatric consultation during hospitalization. However, selected CGA-related components were delivered on an ad hoc basis, including medication reconciliation (75.0%), pharmacist-led medication review (75.0%), early rehabilitation (13.0%), nutritional screening by nutritionists (6.5%), physician-led nutritional management (19.6%), and family counseling by a geriatrician or MDT (3.3%). In contrast, delivery of CGA components was substantially higher in the CGA-consult group, with implementation rates from 78.7% (nutritional screening) to 95.5% (early rehabilitation), and all patients received geriatric consultation (100%). The CGA-ward group demonstrated comprehensive delivery of all CGA components to all participants.

### Clinical outcomes

#### Health-related quality of life

Health-related quality of life showed distinct recovery trajectories among the three groups over the 6-month follow-up period. In the CGA-ward group, mean utility increased substantially from 0.32 at baseline to 0.73 at 1 month, 0.81 at 3 months, and 0.74 at 6 months. In contrast, patients in the usual care and CGA-consult groups showed an initial decline at 1 month (from 0.67 to 0.58 and from 0.68 to 0.65, respectively), followed by partial recovery at 3 months (0.67 and 0.72), but declined again at 6 months (0.56 and 0.55, respectively) (Appendix 5 A and 5B). Analysis of mean changes from baseline demonstrated that CGA-ward patients achieved marked early improvement at 1 month (+ 0.40), with further gains at 3 months (+ 0.47) and sustained benefit at 6 months (+ 0.40). In comparison, the usual care and CGA-consult groups showed minimal early change at 1 month (− 0.02 and + 0.02), followed by modest improvements at 3 months (+ 0.06 and + 0.13) and slightly larger gains by 6 months (+ 0.08 and + 0.15, respectively) (Table 2).

**Table 2:** Mean change of utility and functional scores from baseline for each group

**Utility score**						
Group	1-month		3-month		6-month	
	n	Mean change (SD)	n	Mean change (SD)	n	Mean change (SD)
Usual care	77	-0.02 (0.33)	70	0.06 (0.26)	33	0.08 (0.26)
CGA-consult	77	0.02 (0.35)	69	0.13 (0.31)	31	0.15 (0.25)
CGA-ward	39	0.40 (0.30)	35	0.47 (0.30)	30	0.40 (0.33)
**Functional score**						
Group	1-month		3-month		6-month	
	n	Mean change (SD)	n	Mean change (SD)	n	Mean change (SD)
Usual care	77	-4.7 (25.3)	71	0.1 (23.4)	33	3.8 (25.2)
CGA-consult	81	-2.9 (27.2)	72	4.9 (24.8)	31	8.7 (19.9)
CGA-ward	39	35.4 (27.9)	35	42.6 (27.8)	31	40.0 (29.9)

*Abbreviations*: *CGA *Comprehensive geriatric assessment, *SD *Standard deviation

The mean changes in utility score after adjusting for confounding factors revealed the most significant improvement in the CGA-ward group, with an adjusted mean change of 0.459 (95%CI 0.321, 0.598; *p* < 0.001) (Table 3). The mean change in the CGA-consult group did not differ significantly from the usual care group, with an adjusted mean change of 0.078 (95%CI − 0.026, 0.181; *p* = 0.141).

**Table 3:** Clinical outcomes over 6 months post- discharge: A generalized linear mixed-effects model with inverse-probability treatment weighting

	HRQoL IPTW, Coef. of mean change (95% CI)	p-value	Functional outcomeIPTW, Coef. of mean change (95% CI)	p-value	RehospitalizationAdjusted RR (95% CI)	p-value
Intervention						
Usual care (n = 92)	0		0		1	
CGA-consult (n = 89)	0.078 (-0.026, 0.181)	0.141	3.19 (-4.33, 10.70)	0.406	0.86 (0.58, 1.29)	0.469
CGA-ward (n = 45)	0.459 (0.321, 0.598)	<0.001^a^	37.94 (26.39, 49.48)	<0.001^a^	0.20 (0.09, 0.42)	<0.001^a^
Time point						
1-month visit	0		0		1	
3-month visit	0.095 (0.056, 0.134)	<0.001^a^	7.20 (5.00, 9.41)	<0.001^a^	1.58 (1.21, 2.08)	0.001^a^
6-month visit	0.041 (-0.004, 0.087)	0.075	7.50 (3.79, 11.21)	<0.001^a^	1.49 (1.09, 2.03)	0.012^a^
Potential variables						
CKD stage 3-5	0.080 (-0.015, 0.176)	0.100	0.68 (-5.98, 7.35)	0.841	1.00 (0.67, 1.48)	0.983
Dementia	0.018 (-0.108, 0.145)	0.777	-5.78 (-15.43, 3.88)	0.241	1.03 (0.61, 1.75)	0.900
Baseline Barthel Index	-0.000 (-0.002, 0.002)	0.956	-	-	-	-
Baseline utility score	-	-	-15.47 (-30.35, -0.59)	0.042^a^	0.70 (0.33, 1.46)	0.340

*Abbreviations*: *CGA *Comprehensive geriatric assessment, *CI *Confidence interval, *CKD *Chronic kidney disease, *Coef *Coefficient, *HRQoL*, Health-related quality of life, *IPTW *Inverse probability of treatment weighting, *RR *Relative risk

^a^Indicates statistical significance (<0.05)

In IPTW-adjusted models, the CGA-ward group demonstrated the greatest improvement in utility compared with usual care. This effect was consistent across all time points, with significant improvements at 1 month (mean difference (MD) 0.225, 95% CI 0.117, 0.334; *p* < 0.001), 3 months (MD 0.239, 95% CI 0.128, 0.349; *p* < 0.001), and 6 months (MD 0.259, 95% CI 0.140, 0.378; *p* < 0.001). In contrast, the CGA-consult group showed smaller improvements than usual care. At the time-point level, the difference reached statistical significance at 3 months (MD 0.073, 95% CI 0.001, 0.146; *p* = 0.049) and 6 months (MD 0.093, 95% CI 0.007, 0.179; *p* = 0.033), but not at 1 month (MD 0.060, 95% CI − 0.009, 0.128; *p* = 0.087) (Appendix 6).

#### Functional outcome

Functional scores demonstrated different recovery patterns across groups over the 6-month follow-up. In the CGA-ward group, the mean Barthel index increased markedly from 29.0 at baseline to 66.0 at 1 month, 74.4 at 3 months, and 70.3 at 6 months. In contrast, the usual care and CGA-consult groups showed modest early declines at 1 month (from 75.3 to 70.6 and from 72.6 to 69.9, respectively), followed by gradual recovery at 3 months and slightly higher compared to baseline at 6 months (81.8 and 82.7, respectively) (Appendix 5 A and 5 C).

When expressed as mean changes from baseline, CGA-ward patients showed substantial early improvement at 1 month (+ 35.4), with continued gains at 3 months (+ 42.6) and sustained benefit at 6 months (+ 40.0). By comparison, the usual care and CGA-consult groups experienced small declines at 1 month (− 4.7 and − 2.9), followed by modest improvements at 6 months (+ 3.8 and + 8.7, respectively). Overall, functional recovery occurred in all groups, but the magnitude and timing of improvement were most pronounced in the CGA-ward group (Table 2).

After adjusting for potential confounders, significant improvements in functional changes were identified in the CGA-ward group compared to usual care, with a MD of 37.94 (95% CI 26.39, 49.48; *p* < 0.001). In contrast, there was no significant difference between the CGA-consult and usual care groups, with a MD of 3.19 (95% CI − 4.33, 10.70); *p* = 0.406) (Table 3).

#### Rehospitalization

At 1-month follow-up, the rehospitalization rate in the CGA-ward group was markedly lower than CGA-consult and usual care groups (6.7% vs. 37.7% vs. 43.5%, respectively). At 3 months, the CGA-ward group maintained a lower rehospitalization rate (20.5% vs. 53.6% vs. 49.4%), with a similar pattern at 6 months (19.5% vs. 35.4% vs. 38.5%) (Appendix 7). Adjusting for confounding factors in Poisson regression with IPTW, the CGA-ward intervention significantly reduced rehospitalization risk (adjusted relative risk (aRR) 0.20, 95%CI 0.09, 0.42; *p* < 0.001), while no significant difference was observed for the CGA-consult group (aRR 0.86, 95%CI 0.58, 1.29; *p* = 0.469) (Table 3).

### Resource utilization and cost

At 6 months, total societal costs were lowest for CGA-ward (US$5,916), followed by CGA-consult (US$5,944) and usual care (US$7,446). DMCs were highest in usual care (US$6,800), followed by CGA-consult (US$5,263) and CGA-ward (US$3,946). DNMCs were greatest in the CGA-ward (US$2,285) compared to the CGA-consult (US$758) and usual care (US$831). CGA-ward had the lowest rehospitalization rate (0.6) and lower inpatient, outpatient, and rehospitalization costs (Appendix 8). Workforce costs for CGA-ward were included in DMCs, while CGA-consult was separately estimated at US$28.40 per patient (mainly driven by physical therapist, physician, and pharmacist time) (Appendix 9).

Incremental cost of CGA-consult versus usual-care was − 0.13 (95% CI -0.25, 0.00; *p* = 0.057) and − 0.21(95% CI -0.50, 0.07; *p* = 0.147) for CGA-ward versus usual-care (Appendix 10).

### Cost-effectiveness analysis

Table 4 presents the adjusted cost-utility results. CGA-ward achieved a greater QALY gain of 0.130 with US$937 cost saved, while CGA-consult estimated a QALY gain of 0.047 with US$552 cost saved. The corresponding ICERs were US$-7,208 and US$-11,745 per QALY gained, indicating both interventions were dominant over usual care.

**Table 4:** Adjusted cost-utility analysis

Results	Societal perspective		
	Usual care	CGA-consult	CGA-ward
Total costs, mean (SD) (US$)	7,446 (6,458)	5,944 (6,418)	5,916 (5,254)
Adjusted incremental costs (US$)	Reference	-552	-937
Adjusted incremental QALYs	Reference	0.047	0.130
ICER (US$/QALY gained)	Reference	-11,745^a^	-7,208^a^

*Abbreviations*: *CGA *Comprehensive geriatric assessment, *ICER *Incremental cost-effectiveness ratio, *QALYs *Quality-adjusted life years, *SD *Standard deviation, *US$ *United States Dollar

^a^Dominant indicates that the intervention is more effective and less costly than the comparator

### Uncertainty analysis

**Fig. 1:**
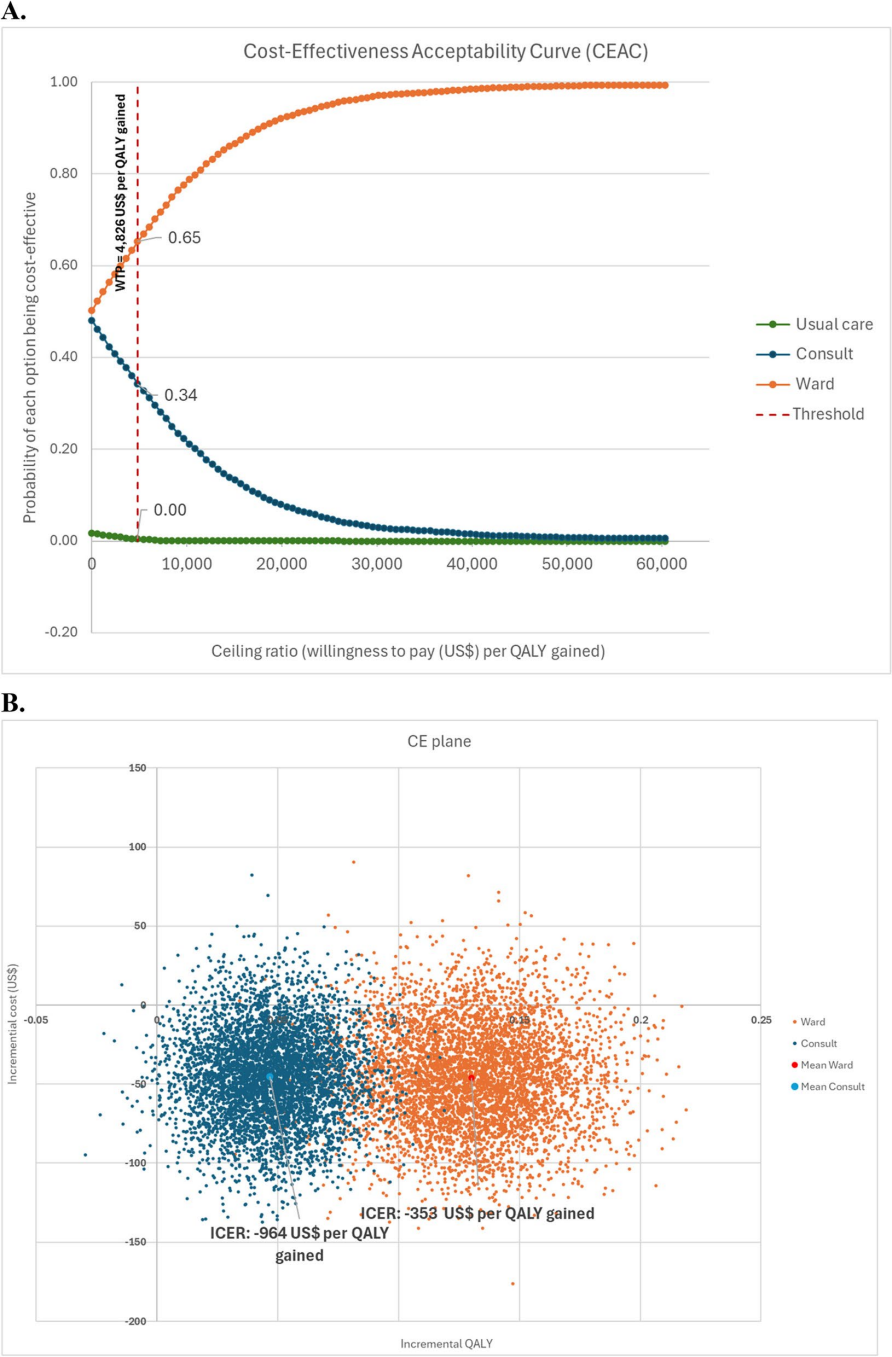
**A** Cost-effectiveness acceptability curve. Abbreviations: CEAC, cost-effectiveness acceptability curve; QALYs, quality-adjusted life years; US$, United States Dollar. **B** Cost-effectiveness plane. Abbreviations: CE, cost-effectiveness; ICER, incremental cost-effectiveness ratio; QALYs, quality-adjusted life years; US$, United States Dollar

Figure 1A shows the CEAC. At the WTP threshold of US$4,826 (THB 160,000), the probability of being cost-effective was highest for the CGA-ward model (65%), followed by the CGA-consult model (34%), while usual care had a negligible probability (approximately 0%). As the WTP increased, the probability of cost-effectiveness for the CGA-ward model continued to rise, whereas that for the CGA-consult model declined. Figure 1B, the CEP, shows that most of the ICER simulations for both CGA models fell in the south-east quadrant, indicating greater QALYs and lower costs. The mean ICERs were US$-353 for CGA-ward and US$-964 for CGA-consult.

## Discussion

This study compared two models of CGA with usual care based on data from two subcohorts that enabled direct comparison of clinical and cost effectiveness in the same setting. The findings demonstrate that the CGA-ward model significantly improved HRQoL, functional outcomes, and reduced hospital readmissions among frail older adults over a 6-month follow-up period. In contrast, the CGA-consult model showed a trend toward improved HRQoL but showed no significant impact on functional score and readmission, highlighting a more limited impact relative to the CGA-ward model. Geriatric care was partially incorporated into usual care, and delivery was fragmented and inconsistent compared with the structured, comprehensive CGA approaches in the CGA-consult and CGA-ward groups. Notably, both CGA approaches were cost-saving, underscoring their value in resource-limited settings.

Our findings aligned with a recent meta-analysis highlighting the benefit of CGA on patients’ HRQoL [18]. Specifically, the CGA-ward model provided the largest benefit of HRQoL in our study. For functional outcomes, an earlier meta-analysis reported no benefit of the CGA model [12], but our study observed improvements in functional outcomes along with HRQoL for the CGA-ward group, highlighting comprehensive benefits. Notably, our study utilized the mean change from baseline for the CGA-ward group, reflecting the selected group of frail older patients whose function deteriorated during acute illnesses gained significant improvement, offering a clearer benefit of CGA effectiveness [32]. Furthermore, our study demonstrated that the CGA-ward model was also associated with a reduction in hospital readmissions similar to that observed previously [33].

The benefits observed for clinical outcomes may reflect the comprehensiveness of the CGA-ward intervention, which addressed multidimensional needs through individualized care plans and caregiver education. Few prior studies have examined caregiver training within CGA [10, 12, 18], with most focusing on social support or discharge planning [12, 18] but not specifically on caregiver training. Among frail older patients, the decline in physical and cognitive function during admission often leads to negative patient outcomes [3, 8], which require close monitoring and ongoing health and social care support during this transition period post-discharge. Appropriate interventions during the hospital-to-home transition period have proven beneficial; however, the effect of integrating them within CGA models has been inconsistent [34]. Our study highlights the potential benefits of CGA-ward on patient outcomes, HRQoL, and cost-effectiveness associated with the inclusion of these care components during the transition period for frail older patients with functional and cognitive impairment.

Our study demonstrated a modest improvement for the CGA-consult model. A previous meta-analysis showed little benefit to HRQoL [20], in support of prior reports suggesting that consultative models, which offer less intensive care, achieve limited improvements in HRQoL [18]. Moreover, many CGA-consult models utilize a care team with limited explicit training on several aspects of supporting frail older patients, possibly lacking sufficient knowledge to identify and address more subtle needs of vulnerable patients [14]. For instance, delirium is a prevalent geriatric syndrome during admission but commonly undiagnosed in general wards [35, 36]. Our findings reaffirm the value of a MDT equipped within a dedicated ward to care for the complex needs of hospitalized frail older patients.

From an economic perspective, both CGA models in our study were more effective and cost-saving compared to usual care. CGA-ward achieved greater QALY gains, offering better value-for-money. A Swedish trial showed CGA-ward improved outcomes and reduced costs [37] in contrast to a UK study, which found that a post-discharge CGA model led to no significant QALY improvement and was therefore not cost-effective [38]. These contrasting findings should be interpreted with caution, as the Swedish CGA-ward included patients with more severe baseline frailty, which may have increased the likelihood of observing more improvements. This underscores the importance of considering not only whether CGA is provided, but also how, when, and in what clinical context it is delivered. Our study addresses locally adapted features—such as caregiver education and community linkage—likely enhanced effectiveness and cost-efficiency. This is especially important in LMICs with limited access to formal post-acute care.

Our study has several strengths. First, it is among the few to comprehensively evaluate both clinical and economic outcomes of two CGA models in the same healthcare setting, providing relevant insight for LMICs. Second, the use of multiple cohorts with pre-specified protocols minimized recall and ascertainment bias. Third, IPTW improved internal validity by adjusting for baseline confounders. Lastly, including caregiver training and discharge planning for the CGA-ward model enhances generalizability and supports localized implementation in resource-limited settings. This study also has several limitations. First, the evaluation combined randomized data for CGA-consult versus usual care with a non-randomized cohort of patients receiving CGA-ward care, resulting in differences in baseline characteristics. Although IPTW and multivariable regression were used to reduce observable imbalances, residual confounding from unmeasured factors cannot be excluded. Therefore, findings related to the CGA-ward model should be interpreted as pragmatic, real-world effectiveness evidence. Second, the single-center university hospital setting may limit the generalizability of the findings. Third, patient selection in the cohort group may have introduced selection bias. Fourth, DMCs were estimated using a top-down approach based on routinely collected administrative data rather than patient-level micro-costing. While this approach may not fully capture individual-level cost heterogeneity, it enabled comprehensive cost capture and reflects real-world expenditure patterns within the healthcare system. To address variability and uncertainty, we applied regression-based adjustment to account for baseline differences, log-transformation for skewed cost data, and PSA, which together strengthen the robustness of cost-effectiveness estimates. Lastly, the number of participants contributing HRQoL and functional data decreased at later follow-up time points. Consequently, some early-enrolled participants did not undergo prospective assessments at 6 months. Because outcomes were collected using standardized prospective instruments, retrospective evaluation was not performed to minimize recall bias and measurement inaccuracy. Although longitudinal mixed-effects models were used to incorporate all available observations and mitigate potential bias related to incomplete repeated measurements, the reduced effective sample size at later time points may have limited statistical precision, particularly for functional and readmission outcomes. Nevertheless, the overall direction and magnitude of outcome trends were broadly consistent with prior studies.

## Conclusions

The CGA-ward model (IMC ward) in our study represents an opportunity to improve patient care in resource-limited settings, demonstrated by significant clinical and economic benefits. While less impactful, the CGA-consult model offers a practical alternative where dedicated wards are unavailable. These findings support the integration of CGA into routine care and offer valuable evidence to inform policy decisions for optimizing the care of hospitalized frail older adults.

## Supplementary Information

Supplementary Material 1.

## Data Availability

The datasets used and/or analyzed in the current study are available from the corresponding author on reasonable request.

## References

[1] Kim DH, Rockwood K. Frailty in older adults. N Engl J Med. 2024;391(6):538–48. 39115063 10.1056/NEJMra2301292 PMC11634188

[2] Hoogendijk EO, Afilalo J, Ensrud KE, Kowal P, Onder G, Fried LP. Frailty: implications for clinical practice and public health. Lancet. 2019;394(10206):1365–75. 31609228 10.1016/S0140-6736(19)31786-6

[3] Cunha AIL, Veronese N, de Melo Borges S, Ricci NA. Frailty as a predictor of adverse outcomes in hospitalized older adults: a systematic review and meta-analysis. Ageing Res Rev. 2019;56:100960. 31518686 10.1016/j.arr.2019.100960

[4] Moreno-Aguilar M, García-Lara JM, Aguilar-Navarro S, Navarrete-Reyes AP, Amieva H, Ávila-Funes JA. The phenotype of frailty and health-related quality of life. J Frailty Aging. 2013;2(1):2–7. 27070451 10.14283/jfa.2013.1

[5] Kojima G, Iliffe S, Jivraj S, Walters K. Association between frailty and quality of life among community-dwelling older people: a systematic review and meta-analysis. J Epidemiol Community Health. 2016;70(7):716–21. 26783304 10.1136/jech-2015-206717

[6] Gómez Jiménez E, Avendaño Céspedes A, Cortés Zamora EB, García Molina R, Abizanda P. Frailty prevalence in hospitalized older adults: a systematic review. Rev Esp Salud Publica. 2021;95:e202107102. 34620820

[7] Srinonprasert V, Chalermsri C, Aekplakorn W. Frailty index to predict all-cause mortality in Thai community-dwelling older population: a result from a National Health Examination Survey cohort. Arch Gerontol Geriatr. 2018;77:124–8. 29751290 10.1016/j.archger.2018.05.002

[8] Suraarunsumrit P, Sinthornkasem P, Petchthai P, Sainimnuan S, Preedachitkul R, Srinonprasert V. Impact of frailty on healthcare utilization in older patients admitted to medical wards: a study from a large medical school in a middle-income setting. Siriraj Med J. 2025;77(1):83–92.

[9] Rezaei-Shahsavarloo Z, Atashzadeh-Shoorideh F, Gobbens RJJ, Ebadi A, Ghaedamini Harouni G. The impact of interventions on management of frailty in hospitalized frail older adults: a systematic review and meta-analysis. BMC Geriatr. 2020;20(1):526. 33272208 10.1186/s12877-020-01935-8 PMC7712609

[10] Parker SG, McCue P, Phelps K, McCleod A, Arora S, Nockels K, et al. What is comprehensive geriatric assessment (CGA)? An umbrella review. Age Ageing. 2018;47(1):149–55. 29206906 10.1093/ageing/afx166

[11] Suraarunsumrit P, Srinonprasert V, Kongmalai T, Suratewat S, Chaikledkaew U, Rattanasiri S et al. Outcomes associated with postoperative cognitive dysfunction: a systematic review and meta-analysis. Age Ageing. 2024;53(7). 10.1093/ageing/afae160 PMC11277860 39058915

[12] Ellis G, Gardner M, Tsiachristas A, Langhorne P, Burke O, Harwood RH, et al. Comprehensive geriatric assessment for older adults admitted to hospital. Cochrane Database Syst Rev. 2017;9:CD006211. 28898390 10.1002/14651858.CD006211.pub3 PMC6484374

[13] Ellis G, Whitehead MA, Robinson D, O’Neill D, Langhorne P. Comprehensive geriatric assessment for older adults admitted to hospital: meta-analysis of randomised controlled trials. BMJ. 2011;343:d6553. 22034146 10.1136/bmj.d6553 PMC3203013

[14] Deschodt M, Flamaing J, Haentjens P, Boonen S, Milisen K. Impact of geriatric consultation teams on clinical outcome in acute hospitals: a systematic review and meta-analysis. BMC Med. 2013;11:48. 23433471 10.1186/1741-7015-11-48 PMC3626668

[15] Ekdahl AW, Sjöstrand F, Ehrenberg A, Oredsson S, Stavenow L, Wisten A, et al. Frailty and comprehensive geriatric assessment organized as CGA-ward or CGA-consult for older adult patients in the acute care setting: a systematic review and meta-analysis. Eur Geriatr Med. 2015;6(6):523–40.

[16] Sum G, Nicholas SO, Nai ZL, Ding YY, Tan WS. Health outcomes and implementation barriers and facilitators of comprehensive geriatric assessment in community settings: a systematic integrative review [PROSPERO registration no.: CRD42021229953]. BMC Geriatr. 2022;22(1):379. 35488198 10.1186/s12877-022-03024-4 PMC9052611

[17] Tan MP. Healthcare for older people in lower and middle income countries. Age Ageing. 2022;51(4):afac028. 35373815 10.1093/ageing/afac016

[18] Chen Z, Ding Z, Chen C, Sun Y, Jiang Y, Liu F, et al. Effectiveness of comprehensive geriatric assessment intervention on quality of life, caregiver burden and length of hospital stay: a systematic review and meta-analysis of randomised controlled trials. BMC Geriatr. 2021;21(1):377. 34154560 10.1186/s12877-021-02319-2 PMC8218512

[19] Counsell SR, Callahan CM, Clark DO, Tu W, Buttar AB, Stump TE, et al. Geriatric care management for low-income seniors: a randomized controlled trial. JAMA. 2007;298(22):2623–33. 18073358 10.1001/jama.298.22.2623

[20] Sriwong WT, Mahavisessin W, Srinonprasert V, Siriussawakul A, Aekplakorn W, Limpawattana P, et al. Validity and reliability of the Thai version of the simple frailty questionnaire (T-FRAIL) with modifications to improve its diagnostic properties in the preoperative setting. BMC Geriatr. 2022;22(1):161. 35227210 10.1186/s12877-022-02863-5 PMC8883653

[21] Charlson ME, Pompei P, Ales KL, MacKenzie CR. A new method of classifying prognostic comorbidity in longitudinal studies: development and validation. J Chronic Dis. 1987;40(5):373–83. 3558716 10.1016/0021-9681(87)90171-8

[22] Albert SM, Bear-Lehman J, Burkhardt A. Lifestyle-adjusted function: variation beyond BADL and IADL competencies. Gerontologist. 2009;49(6):767–77. 19506030 10.1093/geront/gnp064 PMC2775899

[23] Kaiser MJ, Bauer JM, Ramsch C, Uter W, Guigoz Y, Cederholm T, et al. Validation of the Mini Nutritional Assessment short-form (MNA-SF): a practical tool for identification of nutritional status. J Nutr Health Aging. 2009;13(9):782–8. 19812868 10.1007/s12603-009-0214-7 PMC12878690

[24] Herdman M, Gudex C, Lloyd A, Janssen M, Kind P, Parkin D, et al. Development and preliminary testing of the new five-level version of EQ-5D (EQ-5D-5L). Qual Life Res. 2011;20(10):1727–36. 21479777 10.1007/s11136-011-9903-x PMC3220807

[25] Pattanaphesaj J, Thavorncharoensap M, Ramos-Goñi JM, Tongsiri S, Ingsrisawang L, Teerawattananon Y. The EQ-5D-5L valuation study in Thailand. Expert Rev Pharmacoecon Outcomes Res. 2018;18(5):551–8. 29958008 10.1080/14737167.2018.1494574

[26] Nguyen AT, Nguyen LH, Nguyen TX, Nguyen HTT, Nguyen TN, Pham HQ, et al. Frailty prevalence and association with health-related quality of life impairment among rural community-dwelling older adults in Vietnam. Int J Environ Res Public Health. 2019;16(20):3869. 31614836 10.3390/ijerph16203869 PMC6843267

[27] Xu RH, Wong EL, Cheung AW. Estimation of minimally important difference of the EQ-5D-5L utility scores among patients with either hypertension or diabetes or both: a cross-sectional study in Hong Kong. BMJ Open. 2020;10(11):e039397. 33243797 10.1136/bmjopen-2020-039397 PMC7692985

[28] Chesnaye NC, Stel VS, Tripepi G, Dekker FW, Fu EL, Zoccali C, et al. An introduction to inverse probability of treatment weighting in observational research. Clin Kidney J. 2022;15(1):14–20. 35035932 10.1093/ckj/sfab158 PMC8757413

[29] Austin PC. Balance diagnostics for comparing the distribution of baseline covariates between treatment groups in propensity-score matched samples. Stat Med. 2009;28(25):3083–107. 19757444 10.1002/sim.3697 PMC3472075

[30] Mihaylova B, Briggs A, O’Hagan A, Thompson SG. Review of statistical methods for analysing healthcare resources and costs. Health Econ. 2011;20(8):897–916. 20799344 10.1002/hec.1653 PMC3470917

[31] Thavorncharoensap M, Teerawattananon Y, Natanant S, Kulpeng W, Yothasamut J, Werayingyong P. Estimating the willingness to pay for a quality-adjusted life year in Thailand: does the context of health gain matter? Clinicoecon Outcomes Res. 2013;5:29–36. 23345984 10.2147/CEOR.S38062 PMC3548562

[32] Wong YG, Hang JA, Francis-Coad J, Hill AM. Using comprehensive geriatric assessment for older adults undertaking a facility-based transition care program to evaluate functional outcomes: a feasibility study. BMC Geriatr. 2022;22(1):598. 35850671 10.1186/s12877-022-03255-5 PMC9294817

[33] Lin KP, Chen JH, Lu FP, Wen CJ, Chan DD. The impact of early comprehensive geriatric screening on the readmission rate in an acute geriatric ward: a quasi-experimental study. BMC Geriatr. 2019;19(1):285. 31651249 10.1186/s12877-019-1312-y PMC6813968

[34] Liebzeit D, Rutkowski R, Arbaje AI, Fields B, Werner NE. A scoping review of interventions for older adults transitioning from hospital to home. J Am Geriatr Soc. 2021;69(10):2950–62. 34145906 10.1111/jgs.17323 PMC8497409

[35] Ruangratsamee S, Assanasen J, Praditsuwan R, Srinonprasert V. Unrecognized delirium is prevalent among older patients admitted to general medical wards and lead to higher mortality rate. J Med Assoc Thai. 2016;99(8):904–12. 29947497

[36] Titlestad I, Haugarvoll K, Solvang SH, Norekvål TM, Skogseth RE, Andreassen OA, et al. Delirium is frequently underdiagnosed among older hospitalised patients despite available information in hospital medical records. Age Ageing. 2024;53(2):afae020. 38342753 10.1093/ageing/afae006 PMC10859244

[37] Ekerstad N, Karlson BW, Andersson D, Husberg M, Carlsson P, Heintz E, et al. Short-term resource utilization and cost-effectiveness of comprehensive geriatric assessment in acute hospital care for severely frail elderly patients. J Am Med Dir Assoc. 2018;19(10):871–e82. 29784592 10.1016/j.jamda.2018.04.003

[38] Tanajewski L, Franklin M, Gkountouras G, Berdunov V, Edmans J, Conroy S, et al. Cost-effectiveness of a specialist geriatric medical intervention for frail older people discharged from acute medical units: economic evaluation in a two-centre randomised controlled trial (AMIGOS). PLoS ONE. 2015;10(5):e0121340. 25942421 10.1371/journal.pone.0121340 PMC4420253

[39] Ohta R, Sano C. Risk of hospital readmission among older patients discharged from the rehabilitation unit in a rural community hospital: a retrospective cohort study. J Clin Med. 2021;10(4):640. 33572128 10.3390/jcm10040659 PMC7916054

