# Supplementary material for: Clinical outcomes and cost-utility analysis of comprehensive geriatric assessment models in hospitalized frail older patients in Thailand
Source: BMC Geriatr. 2026 Jun 3;26:1013. doi: 10.1186/s12877-026-07718-x (PMC13445659; doi:10.1186/s12877-026-07718-x)
Supplement: Supplementary file 1 [file 12877_2026_7718_MOESM1_ESM.docx]

**Supplementary Material**

**Clinical Outcomes and Cost-Utility Analysis of Comprehensive Geriatric Assessment Models in Hospitalized Frail Older Patients in Thailand**

**Appendix 1. Detailed activity differences between CGA models and usual care**

**Appendix 2. Study flow**

**Appendix 3. Principal diagnosis on admission and selected subcategories**

**Appendix 4. Delivery of CGA components during index hospitalization among study groups**

**Appendix 5. Changes in health-related quality of life and functional status over 6 months**

**Appendix 6. Comparison of HRQoL over 6 months: a multivariate, mixed-effect regression model**

**Appendix 7. Rehospitalization**

**Appendix 8. Unadjusted calculation of costs at 6 months follow-up based on the societal perspective**

**Appendix 9. Costs of the CGA-consult model**

**Appendix 10. Total costs over 6 months: A multivariable log-linear regression model with IPTW**

**Appendix 1. The details of different activities between the CGA models and the usual care**

|  | **Usual care** | **CGA-consult** | **CGA-ward** |
| --- | --- | --- | --- |
| **In-hospital activities** | | | |
| Problem list stratification and management by doctors | **+** | **+** | **+** |
| Mental evaluation and management by nurses | **-** | **+** | **+** |
| Mental intervention by psychologists | **-** | **-** | **+** |
| Medication review with interventions by pharmacists | **+/-** | **+** | **+** |
| Nutritional screening & support by nutritionists | **+/-** | **+** | **+** |
| Early rehabilitation strategy by physical therapists | **+/-** | **+** | **+** |
| Thai Traditional Medicine Therapy by Thai traditional medicine doctors | **-** | **-** | **+** |
| Training a primary caregiver to look after a patient appropriately | **-** | **-** | **+** |
| MDT conference having inputs from all involved team on weekly basis | **-** | **-** | **+** |
| Family counseling with MDT or a geriatrician before discharge | **+/-** | **+** | **+** |
| **After-discharge activities** | | | |
| Hotline telephone call except for weekend/official Line 24 hours | **-** | **+** | **-** |
| Telephone call follow-up by doctors at 2 weeks | **-** | **+** | **-** |
| Telemedicine by a MDT at 1, 3, and 6 months | **-** | **+** | **-** |
| Telephone call follow-up by nurses at 1, 3, and 6 months | **-** | **-** | **+** |
| Regular appointments by primary physicians | **+** | **+** | **+** |

Abbreviations: CGA, comprehensive geriatric assessment; MDT, multidisciplinary team

**Appendix 2. Study Flow**

Hospitalized older patients in local area

(Apr 2023-Jun 2024)

(n = 80)

Hospitalized frail older patients in local area

(Sep 2019-Mar 2024)

(n = 612)

Patients excluded (n = 431):

- Refused participation (n = 247)

- Unavailable for follow-up (n = 161)

- Unable to communicate verbally (n = 23)

Patients excluded (n = 35):

- Transferred to acute medical wards (n = 19)

- Non-frail (n = 16)

**Admission**

Usual care

n = 92

CGA-ward

n = 45

CGA-consult

n = 89

- Loss to Follow-up (n = 5)

- Death (n = 0)

- Withdrawal (n = 1)

**1 month**

- Loss to Follow-up (n = 3)

- Death (n = 4)

- Withdrawal (n = 3)

- Loss to Follow-up (n = 3)

- Death (n = 6)

- Withdrawal (n = 5)

Evaluate all outcomes

n = 39

Evaluate all outcomes

n = 79

Evaluate all outcomes

n = 78

- Loss to Follow-up (n = 3)

- Death (n = 1)

- Withdrawal (n = 0)

- Loss to Follow-up (n = 2)

- Death (n = 3)

- Withdrawal (n = 2)

- Loss to Follow-up (n = 2)

- Death (n = 6)

- Withdrawal (n = 1)

**3 months**

Evaluate all outcomes

n = 71

Evaluate all outcomes

n = 70

Evaluate all outcomes

n = 35

- Evaluate only rehospitalization (n = 37)^†^

- Death (n = 1)

- Withdrawal (n = 0)

- Evaluate only rehospitalization (n = 36)^†^

- Death (n = 3)

- Withdrawal (n = 0)

- Loss to Follow-up (n = 3)

- Death (n = 0)

- Withdrawal (n = 0)

**6 months**

Evaluate all outcomes

n = 32

Evaluate all outcomes

n = 31

Evaluate all outcomes

n = 33

Abbreviations: CGA, comprehensive geriatric assessment; MDT, multidisciplinary team; OPD, outpatient department; QoL, quality of life

^†^Before the protocol amendment extending the follow-up period to 6 months, QoL and functional outcome data at 3 months were collected from 16% of participants: 36 patients in the CGA-consult group and 37 patients in the control group.

# **Appendix 3. Principal diagnosis on admission and selected subcategories**

| **Diagnosis category** | **Usual care**  **n=92** | **CGA-consult**  **n=89** | **CGA-ward**  **n=45** |
| --- | --- | --- | --- |
| Infection, n (%) | 36 (39.1) | 33 (37.1) | 7 (15.6) |
| Respiratory tract infection | 3 (3.3) | 11 (12.4) | 5 (11.2) |
| Urinary tract infection | 11 (12.0) | 6 (6.7) | 1 (2.2) |
| Other infection | 22 (23.8) | 16 (18.0) | 1 (2.2) |
| Non-infectious conditions, n (%) | 56 (60.9) | 54 (62.1) | 38 (84.4) |
| Congestive heart failure | 17 (18.5) | 18 (20.2) | 0 (0.0) |
| Acute coronary syndrome | 6 (6.5) | 4 (4.5) | 0 (0.0) |
| Acute stroke | 2 (2.2) | 1 (1.1) | 0 (0.0) |
| Rehabilitation-related | 0 (0.0) | 0 (0.0) | 38 (84.4) |
| Medical deconditioning rehabilitation | 0 (0.0) | 0 (0.0) | 12 (26.7) |
| Post-orthopedic/surgical rehabilitation | 0 (0.0) | 0 (0.0) | 20 (44.4) |
| Post-stroke rehabilitation | 0 (0.0) | 0 (0.0) | 6 (13.3) |
| Gastrointestinal disease | 6 (6.5) | 8 (9.2) | 0 (0.0) |
| Nephrological disease | 9 (9.8) | 4 (4.6) | 0 (0.0) |
| Others | 19 (20.7) | 14 (16.1) | 0 (0.0) |

Abbreviations: CGA, comprehensive geriatric assessment

**Appendix 4. Delivery of CGA components during index hospitalization among study groups**

| **CGA component** | **Usual care**  **n=92** | **CGA-consult**  **n=89** | **CGA-ward**  **n=45** |
| --- | --- | --- | --- |
| Any geriatric consultation | 3 (3.3) | 89 (100.0) | 45 (100.0) |
| Medication reconciliation | 69 (75.0) | 80 (89.9) | 45 (100.0) |
| Medication review with interventions by pharmacists | 69 (75.0) | 82 (92.1) | 45 (100.0) |
| Early rehabilitation strategy by physical therapists | 12 (13) | 85 (95.5) | 45 (100.0) |
| Nutritional screening and advice by nutritionists | 6 (6.5) | 70 (78.7) | 45 (100.0) |
| Nutritional management by physicians | 18 (19.6) | 79 (88.8) | 45 (100.0) |
| Family counseling with MDT or a geriatrician before discharge | 3 (3.3) | 82 (92.1) | 45 (100.0) |

Abbreviations: CGA, comprehensive geriatric assessment; MDT, multidisciplinary team

**Appendix 5. Changes in health-related quality of life and functional status over 6 months**

**5A. Actual mean of utility and functional scores**

| **Utility score** | | | | | | | | |
| --- | --- | --- | --- | --- | --- | --- | --- | --- |
| **Group** | **Baseline** | | **1-month** | | **3-month** | | **6-month** | |
|  | **n** | **Mean (SD)** | **n** | **Mean (SD)** | **n** | **Mean (SD)** | **n** | **Mean (SD)** |
| **Usual care** | 90 | 0.67 (0.28) | 87 | 0.58 (0.35) | 81 | 0.67 (0.32) | 46 | 0.56 (0.39) |
| **CGA-consult** | 85 | 0.68 (0.25) | 84 | 0.65 (0.34) | 79 | 0.72 (0.34) | 47 | 0.55 (0.43) |
| **CGA-ward** | 44 | 0.32 (0.26) | 39 | 0.73 (0.24) | 36 | 0.81 (0.25) | 33 | 0.74 (0.29) |
| **Functional score** | | | | | | | | |
| **Group** | **Baseline** | | **1-month** | | **3-month** | | **6-month** | |
|  | **n** | **Mean (SD)** | **n** | **Mean (SD)** | **n** | **Mean (SD)** | **n** | **Mean (SD)** |
| **Usual care** | 91 | 75.3 (24.6) | 78 | 70.6 (28.4) | 72 | 78.0 (26.5) | 33 | 81.8 (25.9) |
| **CGA-consult** | 86 | 72.6 (26.4) | 82 | 69.9 (29.1) | 73 | 78.9 (24.0) | 31 | 82.7 (20.5) |
| **CGA-ward** | 45 | 29 (22.7) | 39 | 66.0 (26.4) | 35 | 74.4 (26.5) | 33 | 70.3 (29.5) |

Abbreviations: CGA, comprehensive geriatric assessment; SD, standard deviation

**5B. Utility scores at different timepoints 5C. Functional scores at different timepoints**

 Abbreviations: CGA, comprehensive geriatric assessment Abbreviations: CGA, comprehensive geriatric assessment

**Appendix 6. Comparison of HRQoL over 6 months: a multivariate, mixed-effect regression model**

|  | **Multivariate analysis**  **Applying IPTW, coef. of actual mean (95% CI)** | | | | | |
| --- | --- | --- | --- | --- | --- | --- |
|  | **1-month** | **p-value** | **3-month** | **p-value** | **6-month** | **p-value** |
| Usual care (n = 92) | 0.006 (-0.002, 0.016) | 0.139 | 0.020 (-0.007, 0.047) | 0.139 | 0.040 (-0.013, 0.094) | 0.139 |
| CGA-consult (n = 89) | 0.060 (-0.009, 0.128) | 0.087 | 0.073 (0.001, 0.146) | 0.049^*^ | 0.093 (0.007, 0.179) | 0.033 |
| CGA-ward (n = 45) | 0.225 (0.117, 0.334) | <0.001^*^ | 0.239 (0.128, 0.349) | <0.001^*^ | 0.259 (0.140, 0.378) | <0.001^*^ |

**Abbreviations:** Coef., coefficient; CI, confidence interval; HRQoL, health-related quality of life; IPTW, inverse probability of treatment weighting. † Adjusted for age, congestive heart failure, chronic kidney disease stage 3–5, dementia, and baseline Barthel Index for Activities of Daily Living (BADL). *Indicates statistical significance (p ≤ 0.05).

**Appendix 7. Rehospitalization**

| **Group** | **1-month** | | **3-month** | | **6-month** | |
| --- | --- | --- | --- | --- | --- | --- |
|  | **n** | **n (%)** | **n** | **n (%)** | **n** | **n (%)** |
| **Usual care** | 85 | 37 (43.5) | 83 | 41 (49.4) | 78 | 30 (38.5) |
| **CGA-consult** | 85 | 32 (37.7) | 84 | 45 (53.6) | 79 | 28 (35.4) |
| **CGA-ward** | 45 | 3 (6.7) | 44 | 9 (20.5) | 41 | 8 (19.5) |

Abbreviations: CGA, comprehensive geriatric assessment

| **Group** | **6-month** | |
| --- | --- | --- |
|  | **n** | **Times, median (min, max)** |
| **Usual care** | 85 | 1 (0, 10) |
| **CGA-consult** | 85 | 1 (0, 9) |
| **CGA-ward** | 45 | 0 (0, 3) |

Abbreviations: CGA, comprehensive geriatric assessment

**Appendix 8. Unadjusted calculation of costs at 6 months follow-up based on the societal perspective**

| **Subcategory** | **Usual care** | | | **CGA-consult** | | | **CGA-ward** | | |
| --- | --- | --- | --- | --- | --- | --- | --- | --- | --- |
|  | n | Mean  (SD) | Median  (min-max) | n | Mean  (SD) | Median  (min-max) | n | Mean  (SD) | Median  (min-max) |
| **Resource utilization underlying direct medical costs** | | | | | | | | | |
| LOS at enrollment, days | 92 | 16  (40) | 7  (2-370) | 89 | 12  (26) | 8  (2-244) | 45 | 22  (10) | 21  (7-54) |
| Number of rehospitalization^†^, times | 85 | 2  (2) | 1  (0-10) | 85 | 1.8  (1.9) | 1  (0-9) | 45 | 0.6  (0.9) | 0  (0-3) |
| Number of OPD visits^†^, times/6 months | 73 | 8  (10) | 4  (0-50) | 67 | 6  (9) | 3  (0-52) | 45 | 12  (10) | 9  (0-45) |
| Duration at OPD visits^†^, hours/6 months | 73 | 26  (57) | 0  (0-366) | 67 | 27  (51) | 0  (0-252) | 45 | 49  (51) | 0  (36-188) |
| **Direct medical cost,** US$/patient | | | | | | | | | |
| Inpatient care costs, US$ | 90 | 2,798 (3,905) | 1,360  (270-24,230) | 87 | 2,041 (1,858) | 1,481  (308-8,227) | 37 | 2,583  (1,329) | 2,361  (874-6,163) |
| Outpatient care costs, US$ | 84 | 1,969 (2,244) | 1,323  (0-12,594) | 81 | 1,670 (1,956) | 1,187  (24-12,045) | 42 | 1,439  (1,797) | 1,016  (74-11,506) |
| Rehospitalization costs, US$ | 81 | 2,207 (4,531) | 0  (0-23,581) | 78 | 1,845 (5,210) | 0  (0-39,478) | 42 | 148  (581) | 0  (0-3,049) |
| Total direct medical costs, US$ | 90 | 6,800 (6,181) | 4,700  (305-31,249) | 87 | 5,263 (5,947) | 3,423  (764-44,613) | 43 | 3,946  (2,264) | 3,509  (176-13,904) |
| **Resource utilization underlying direct non-medical costs** | | | | | | | | | |
| Family caregiving hours^†^ ,hours | 73 | 90  (218) | 7  (0-1,704) | 67 | 80  (163) | 12  (0-1,086) | 45 | 185  (241) | 75  (0-876) |
| **Direct non-medical cost,** US$/patient | | | | | | | | | |
| Transportation costs, US$ | 80 | 86  (133) | 24  (0-741) | 85 | 78  (137) | 21  (0-739) | 26 | 223  (142) | 186  (21-618) |
| Meal expenses, US$ | 80 | 77  (126) | 27  (0-700) | 85 | 50  (109) | 14  (0-793) | 26 | 71  (114) | 3  (0-403) |
| Medications and nutritional supplements, US$ | 80 | 34  (101) | 0  (0-668) | 85 | 31  (74) | 0  (0-398) | 26 | 179  (272) | 11  (0-905) |
| Medical equipment and home modifications, US$ | 80 | 159  (529) | 0  (0-3,017) | 85 | 168  (655) | 0  (0-4,917) | 26 | 365  (521) | 38  (0-1,931) |
| Paid caregiving services, US$ | 80 | 78  (248) | 0  (0-1,388) | 85 | 113  (359) | 0  (0-2,262) | 26 | 375  (1,360) | 0  (0-5,973) |
| Opportunity cost, US$ | 83 | 260  (588) | 62  (0-4,407) | 85 | 208  (430) | 31  (0-2,809) | 26 | 855  (554) | 932  (0-1,981) |
| Total direct non-medical costs, US$ | 70 | 831  (1,039) | 455  (5-4,407) | 78 | 758 (1,149) | 250  (1-5,204) | 26 | 2,285  (1,976) | 1,572  (158-7,564) |
| **Total costs, US$** | 90 | 7,446 (6,458) | 5,237  (305-31,249) | 87 | 5,944 (6,418) | 4,142  (806-48,292) | 43 | 5,916  (5,254) | 4,255  (1,138-32,647) |

Abbreviations: CGA, comprehensive geriatric assessment; LOS, length of stay; OPD, outpatient department; SD, standard deviation; US$, United States Dollar

^†^ The analysis included both users and non-users of resources.

**Appendix 9. Costs of the CGA-consult model**

| Activity | Interaction duration (min/patient) | Cost rate (US$/min) ^†^ | Cost per patient (US$) |
| --- | --- | --- | --- |
| **Initial Assessment** | | | |
| Geriatrician | 15 | 0.13 | 1.99 |
| Pharmacist | 18 | 0.11 | 1.95 |
| Nurse | 15 | 0.10 | 1.51 |
| Nutritionists | 20 | 0.06 | 1.24 |
| Physical therapist | 30 | 0.06 | 1.81 |
| **Ward Round** | | | |
| Geriatrician | 15 | 0.13 | 1.99 |
| Pharmacist | 10 | 0.11 | 1.08 |
| Nurse | 10 | 0.10 | 1.01 |
| Nutritionists | 19 | 0.06 | 1.18 |
| Physical therapist | 86 | 0.06 | 5.18 |
| **Official Line 24 hours** | | | |
| Geriatrician | 15 | 0.13 | 1.99 |
| **Phone calls for 2 weeks** | | | |
| Geriatrician | 15 | 0.13 | 1.99 |
| **Telemedicine** | | | |
| Geriatrician | 10 | 0.13 | 1.33 |
| Pharmacist | 15 | 0.11 | 1.62 |
| Nurse | 10 | 0.10 | 1.01 |
| Nutritionists | 10 | 0.06 | 0.62 |
| Physical therapist | 15 | 0.06 | 0.90 |
| **SUM** | | | **28.40** |

Abbreviations: Min, minute; US$, United States Dollar

^†^Cost rates were calculated based on the actual wages and the interaction duration of each discipline, with all professionals having more than 5 years of experience in caring for older patients.

**Appendix 10. Total costs over 6 months: A multivariable log-linear regression model with IPTW**

|  | **Univariate analysis**  **Log coef. (95% CI)** | **p-value** | **Multivariable analysis**  **Applying IPTW**  **Log coef. (95% CI)** | **p-value** |
| --- | --- | --- | --- | --- |
| **Intervention** | | |  |  |
| Control (n = 90) | 1 |  | 1 |  |
| CGA-consult (n = 87) | -0.18 (-0.30, -0.06) | 0.005* | -0.13 (-0.25, 0.00) | 0.057 |
| CGA-ward (n = 43) | -0.06 (-0.21, 0.09) | 0.408 | -0.21 (-0.50, 0.07) | 0.147 |
|  | | |  |  |
| Age | -0.01 (-0.01, 0.00) | 0.027* | - | - |
| BMI | -0.00 (-0.01, 0.01) | 0.892 | - | - |
| MNA-SF | -0.00 (-0.01, 0.01) | 0.939 | - | - |
| Frailty score | -0.02 (-0.12, 0.08) | 0.679 | - | - |
| CCI | -0.00 (-0.03, 0.02) | 0.691 | - | - |
| HT | -0.14 (-0.31, 0.02) | 0.093 | - | - |
| Type2 DM | 0.06 (-0.06, 0.17) | 0.340 | - | - |
| CHF | 0.10 (-0.02, 0.22) | 0.117 | - | - |
| CAD | -0.10 (-0.23, 0.03) | 0.135 | - | - |
| Stroke or TIA | -0.07 (-0.20, 0.05) | 0.242 | - | - |
| CKD stage 3-5 | 0.05 (-0.07, 0.16) | 0.429 | 0.14 (0.01, 0.26) | 0.035* |
| Dementia | -0.25 (-0.40, -0.10) | 0.001* | -0.07 (-0.24, 0.10) | 0.433 |
| Baseline BADL | -0.00 (-0.00, 0.00) | 0.549 | - | - |
| Baseline EQ-5D-5L | -0.06 (-0.28, 0.15) | 0.560 | - | - |
| Insurance  UC  Other | -0.25 (-0.39, -0.12)  -0.10 (-0.24, 0.03) | <0.001*  0.129 | -  **-** | -  **-** |

Abbreviations: BADL, Barthel Index for activities of daily living; BMI, body mass index; CCI, Charlson comorbidity index; CGA, comprehensive geriatric assessment; CAD, coronary artery disease; CHF, congestive heart failure; CI, confidence interval; CKD, chronic kidney disease; Coef., coefficient; HT, hypertension; IPTW, Inverse probability of treatment weighting; MNA-SF, Mini Nutritional Assessment Short-Form; TIA, transient ischemic attack; Type2 DM, type2 diabetes; UC, Universal Coverage

* Indicates statistical significance (≤0.05)
